# METTL3 expression is associated with glycolysis metabolism and sensitivity to glycolytic stress in hepatocellular carcinoma

**DOI:** 10.1002/cam4.2918

**Published:** 2020-02-18

**Authors:** Ye Lin, Xiangling Wei, Zhixiang Jian, Xuewen Zhang

**Affiliations:** ^1^ Department of Hepatobiliary‐Pancreatic Surgery China‐Japan Union Hospital of Jilin University Changchun Jilin China; ^2^ Department of General Surgery Guangdong Provincial People's Hospital Guangdong Academy of Medical Sciences Guangzhou China

**Keywords:** glycolysis; metaboliosm, Hepatocellular carcinoma, METTL3

## Abstract

METTL3 is an RNA methyltransferase implicated in the control of cell differentiation and proliferation in embryonic development and cancer. The current study was aimed to explore the function and underlying mechanism of METTL3 in hepatocellular carcinoma (HCC). We evaluated the expression and prognostic significance of METTL3 in 100 HCC cases and TCGA dataset. In HCC cases, both the RNA and protein expression of METTL3 were significantly upregulated and associated with poor prognosis. Gene set enrichment analysis of transcriptional profiles in HCC specimens revealed that METTL3 expression was associated with impaired glucose metabolism and mTOR signal pathway. In Huh‐7 and SMMC‐7721 HCC cells, downregulation of METTL3 by siRNA interference inhibited glycolytic capacity, which was proved by the decreased intracellular glucose uptake and lactate production. In terms of mechanism, we found mTORC1 activity was impaired by downregulation of METTL3, additional silencing of METTL3 cannot further decrease the phosphorylation level of mTORC1 and glycolysis activity in Rapamycin‐treated HCC cells. At last, we observed that downregulation of METTL3 synergizes with the glycolysis inhibitor 2‐deoxyglucose (2‐DG) to inhibit tumor growth in vitro. Our study provided evidence that METTL3 is involved in the regulation of glycolysis activity in HCC, suggesting that suppression of glycolysis via METTL3 inhibition was a potential treating strategy against HCC.

## Graphical abstract

This article is the first to elucidate the relationship between METTL3 and glycometabolism in hepatocellular carcinoma, which provides new insight into the role of m6A‐related genes in hepatocellular caricnoma.

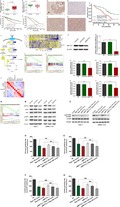

## 1 INTRODUCTION

Cancer is known to be a disease characterized by complex molecular mechanisms driven by oncogenes and oncogenic mutations.^1^, ^2^ The past years have seen great progress toward a deeper understanding of the molecular changes, and emerging evidence demonstrates that metabolic reprogramming is a typical characteristic of cancers. In contrast to normal cells, cancer cells reprogrammed metabolism pathways to fulfill the biosynthetic demands associated with infinite proliferation.^3^ Intriguingly, a markedly increased glucose consumption and enhanced glycolysis facilitating lactate production are observed in tumor cells even under normoxic conditions. This so‐called “Warburg” effect has been identified as a hallmark in distinct tumor types and shown to correlate with tumorigenesis and poor tumor prognosis.^4^

Alterations of hepatic metabolism are critical to the development of liver disease. Hepatocellular carcinoma (HCC) cells showed distinct metabolism pattern from normal hepatocytes and express different metabolic enzymes. Anti‐HCC strategies targeting reprogrammed glucose metabolism attracted lots of interest in recent years.^5^, ^6^ Therefore, identification of novel molecular markers which are associated with metabolism pattern of HCC may be helpful for the treatment of this disease. The methyltransferase‐like 3 (METTL3) is the key component of m6A methyltransferase complex, has shown fundamental significance in diverse biological processes, such as spermatogenesis, embryogenesis, and neurodevelopment.^7^, ^8^, ^9^ Consistent with its important biological function, METTL3 is emerging as a key molecular affecting tumorigenesis cancer progression in a variety of cancers,^10^ including HCC,^11^ but few studies have made attempt to elucidate the relationship between METTL3 and glucose metabolism in HCC. In the current study, the association between METTL3 and glucose metabolism in HCC samples were investigated. A significant positive correlation between METTL3 expression and the transcriptional activity of key genes specific to glycolysis was discovered. Inhibited METTL3 expression sensitizes HCC cells to glycolytic stress. Thereafter, we revealed that mTORC1 signal pathway was implicated in the relationship between METTL3 and glycolysis.

## 2 METHODS

### 2.1 Clinical tissue samples

Between Jan 2013 and December 2013, we obtained 100 tumor tissues and the paired adjacent normal liver tissues from patients who had underwent hepatectomies at our center. Tissue samples were put in liquid nitrogen immediately after surgical resection and then kept in −80°C. HCC was diagnosed by pathological examination. Formalin‐fixed paraffin‐embedded primary specimens were obtained from all patients. The histological types and grades of all samples were determined by experienced pathologists. The study was approved by the Ethical Committee of Guangdong General Hospital. Informed consent was signed by all patients.

### 2.2 Data resource

The level‐3 expression data (HTS eq‐FPKM‐UQ and HTSeq‐Counts), clinicopathological data, and prognosis information of 373 HCC patients and 50 normal liver samples were downloaded from The Cancer Genome Atlas (TCGA, https://tcga-data.nci.nih.gov/tcga/) data portal. EdgeR package in Bioconductor of R language was used to preprocess the normalization of HTSeq‐Counts data.

### 2.3 Immunohistochemistry

Slides (4‐μm thick) of paraffin‐embedded specimens were used for immunohistochemical staining. Briefly, immunochemistry for METTL3 (Rabbit monoclonal; no. ab195352, Abcam Inc) was performed. Sections were baked and de‐paraffinized in xylene and rehydrated in a graded ethanol series, and then treated with 0.3% hydrogen peroxide for 10 minutes, followed by antigen retrieval in 0.01 M citrate buffer (p H: 6.0, 10×). After 20 minutes cooling, sections were incubated with the primarily METTL3 antibody overnight at 4°C. After phosphate‐buffered saline rinses, sections were stained with horseradish peroxidase‐labeled secondary antibody and then were visualized.

### 2.4 Cell culture and reagents

Human hepatocellular carcinoma cells Huh‐7 and SMMC‐7721 were obtained from the Chinese Academy of Sciences Cell Bank. All hepatocellular carcinoma cells were cultured in Dulbecco's modified Eagle's medium (DMEM) (Gibco) supplemented with 10% fetal bovine serum (FBS) (HyClone, South Logan), 100 units/mL penicillin, and 100 μg/mL streptomycin, incubated at 37°C in 5% CO2. 2‐DG was purchased from Sigma.

### 2.5 RNA extraction and real‐time PCR

Total RNA was isolated from cell lines or fresh frozen tissues using the Trizol reagent (TaKaRa, Otsu) according to the manufacturer's protocol. qRT‐PCR was performed using PrimeScriptTM RT reagent Kit (TaKaRa, Otsu). 18sRNA was used as internal control. Relative mRNA expression levels were calculated by the ΔCt method: ΔCt = Ct (targeting gene)‐Ct (18sRNA).

### 2.6 Western blotting

Total protein was extracted in RIPA buffer containing protease inhibitors. The cell lysate used for cell signaling detection was obtained by IP cell lysis solution (Beyotime Biotechnology). Proteins were separated by SDS­PAGE and transferred to a PVDF membrane. The membrane was blocked by 5% bovine serum albumin and then incubated with primary antibody and β‐actin was used as internal control. HRP‐labeled secondary antibodies were used after the gels were incubated with primary antibodies at 4°C overnight. The bands were visualized using the ECL method with Pierce ECL Western Blotting Substrate (Thermo Scientific).

### 2.7 Transient transfection of HCC cells

Cells were plated in six‐well plates at a density of 2 × 105 cells per well. When the cells were in the log phase, specific small interfering RNA (siRNA) targeting METTL3 and the scramble nonsense siRNA which was used as internal control were transfected into cells with Lipofectamine 2000 (Invitrogen, Carlsbad) according to the manufacturer's instruction.

### 2.8 Measurement of glycolytic capacity

Glycolytic ability was measured using a Se‐ ahorse XF96 analyzer (Seahorse Biosciences). Briefly, cells were seeded at 20,000 cells/well in a 94‐well cell culture XF microplate (Seahorse Biosciences). HCC cells were washed and incubated with assay medium for 1 hour at 37°C in a CO2‐free incubator. Plates were then transferred to the XF24 analyzer. All measurements were recorded at set time intervals and normalized to total protein content. ECAR after treatment indicates glycolytic capacity.

### 2.9 CCK‐8 assay

Cell viabilities of HCC cell lines were measured using a CCK‐8 method. Briefly, cells were plated in 96‐well plates at a density of 3000 cells/well, 10 μL CCK‐8 solution was added to the cultures at different time point and incubated at 37°C for 1 hour. The OD values at 450 nm of different treatments were detected and normalized as relative cell viability compared to control groups.

### 2.10 Gene set enrichment analysis

The gene set enrichment analysis (GSEA) was performed according the detailed protocol which is available on the Broad Institute Gene Set Enrichment Analysis website (http://software.broadinstitute.org/gsea). Java GSEA Desktop Application software (V3.0, Broad Institute) was used.

### 2.11 Statistical analysis

We analyzed the data with SPSS 21.0 (SPSS Inc). The differences in gene expression between groups were compared by Student's t‐test. The co‐expression between two genes was analyzed by Spearman's rank correlation test. Kaplan‐Meier method with log‐rank test was used for survival analysis. *P* values less than .05 indicate statistical significance.

## 3 RESULTS

### 3.1 Expression of METTL3 RNA methyltransferase was upregulated in HCC tissues and correlated with prognosis

**Figure 1:**
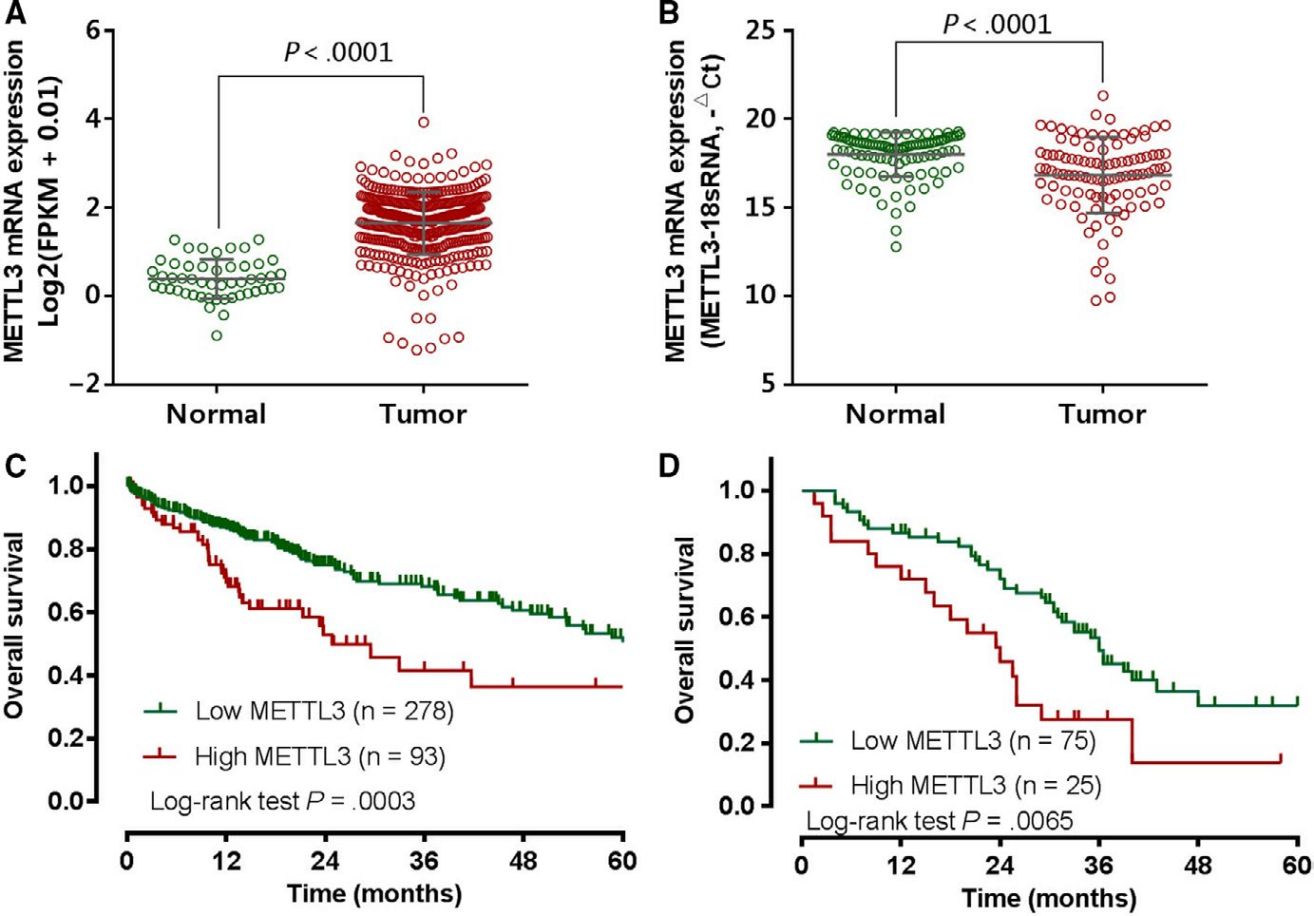
Expression of METTL3 and its prognosis significance in hepatocellular carcinoma. A, METTL3 expression was significantly up‐regulated in HCC tissues in compared with normal liver tissues in TCGA data. B, METTL3 expression was significantly upregulated in HCC tissues in compared with paired adjacent normal liver tissues in patients of our center. Kaplan‐Meier OS curve based on METTL3 expression in TCGA data (C) and patients of our center (D) revealed that high METTL3 expression was associated with poor OS

We analyzed mRNA levels of METTL3 in tumor tissues and normal liver tissues from two independent cohorts. In the TCGA dataset of 50 normal liver samples and 372 HCC samples, we found that METTL3 was significantly upregulated in HCC samples (P ＜ 0.0001) (Figure 1A). To confirm the results, we analyzed METTL3 mRNA levels in 100 cases of HCC patients who underwent hepatectomy at our center. We identified that the level of METTL3 was significantly increased in HCC tissues compared with the corresponding para‐tumor normal liver tissues (*P* ＜ .0001) (Figure 1B). Kaplan‐Meier survival of TCGA data showed significantly worse OS (*P* = .0003) for the METTL3 high‐expression group compared with the low‐expression group (Figure 1C). Consistent with the TCGA cohort, higher METTL3 expression was significantly associated with poorer OS (*P* = .0065) in the 100 patients at our center (Figure 1D).

**Figure 2:**
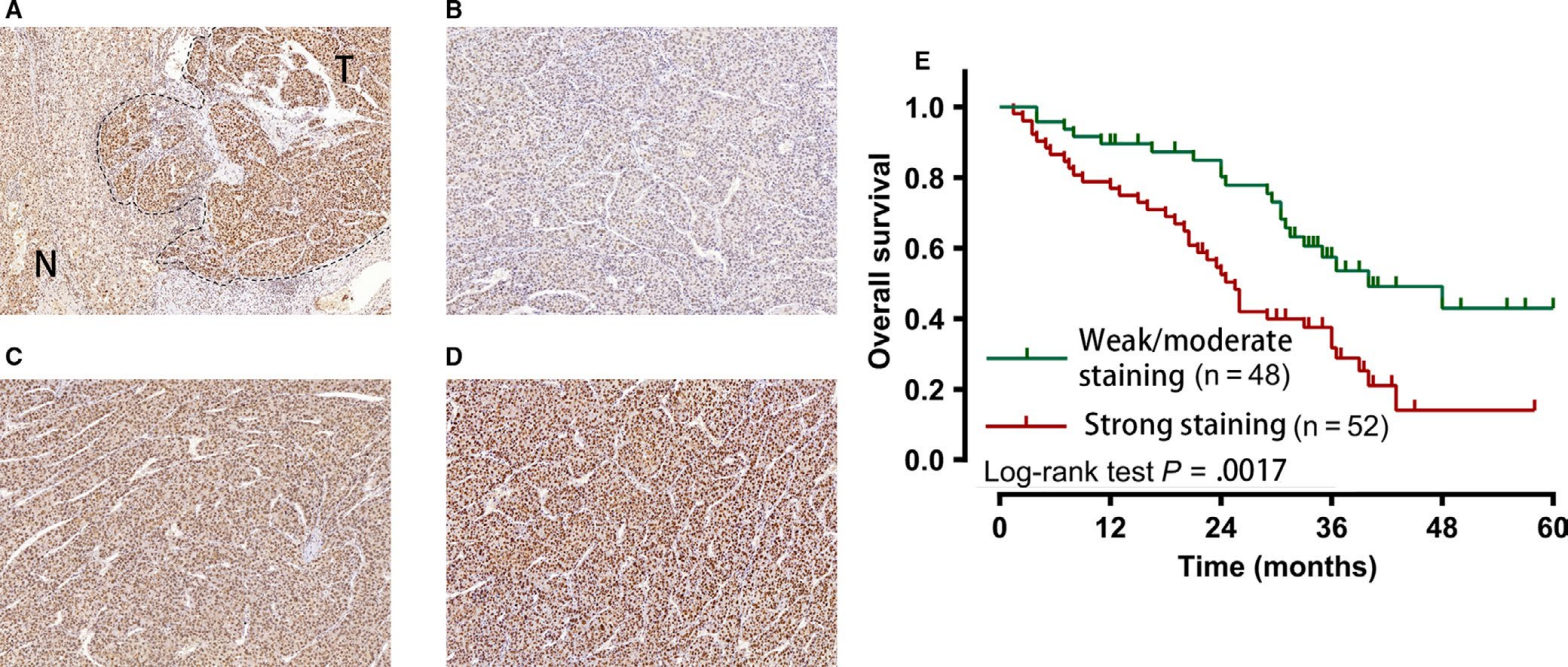
Kaplan‐Meier OS curves for immunohistochemistry of METTL3 in 100 HCC patients. A, Immunohistochemistry staining of METTL3 in HCC tumor tissues and the adjacent normal tissues. (B‐D) Representative images of METTL3 expression in HCC tissues. B, Tissue with weak METTL3 staining. C, Tissue with moderate METTL3 staining. D, Tissue with strong METTL3 staining. E, Comparison of overall survival in patients with HCC with strong METTL3 staining and weak or moderate METTL3 staining. T, tumor tissues; N, normal tissues

We further investigate the expression of METTLE protein in HCC, we conducted immunohistochemical study in cases from our center. The immunostaining of METTL3 was mainly found in the nuclear METTL3 were expressed in both HCC tumor cells and normal liver cells (Figure 2A), 91 (91.0%) cases showed higher METTL3 staining in tumors areas than that in normal liver tissues. Overall, all cases were positive for METTL3 protein in different degrees (Figure 2B‐D), 52 of 100 (52.0%) cases showed strong positive staining for METTL3 protein, 29 (29.0%) cases showed moderate positive staining, and 19 (19.0%) cases had weak METTL3 protein expression. The relationship between METTL3 staining levels and clinicopathological factors are summarized in Table 1. We found that stronger METTL3 staining was correlated with higher histological grading (*P* = .028). Further survival analysis by Kaplan‐Meier method and log‐rank test revealed that patients with strong expression of METTL3 protein had significantly decreased overall survival than those with moderate or weak expression (*P* = .0017).

**Table 1:** Correlation between METTL3 expression and clinicopathological characteristics in 100 cases from our center

Characters	Weak/moderate staining N = 48	Strong staining N = 52	P values
Age, year			
≥50	30	28	0.422
＜50	18	24	
Gender			
Male	32	32	0.678
Female	16	20	
Etiology			
HBV/HCV	36	37	0.822
No‐viral	12	15	
T stage			
T1/T2	33	27	0.104
T3/T4	15	25	
Grade			
G1/G2	30	20	0.028
G3/G4	18	32	
Cirrhosis			
Yes	28	29	0.842
No	20	23	
TNM staging			
I/II	33	26	0.069
III/IV	15	26	
AFP			
>200 μg/L	22	31	0.229
≤200 μg/L	26	21	

Abbreviations: HBV, Hepatitis B Virus; HCV, Hepatitis C Virus; AFP, alpha‐fetoprotein.

TNM staging, the AJCC 8th edition.

### 3.2 METTL3 expression level was associated with altered glucose metabolism in HCC

**Figure 3:**
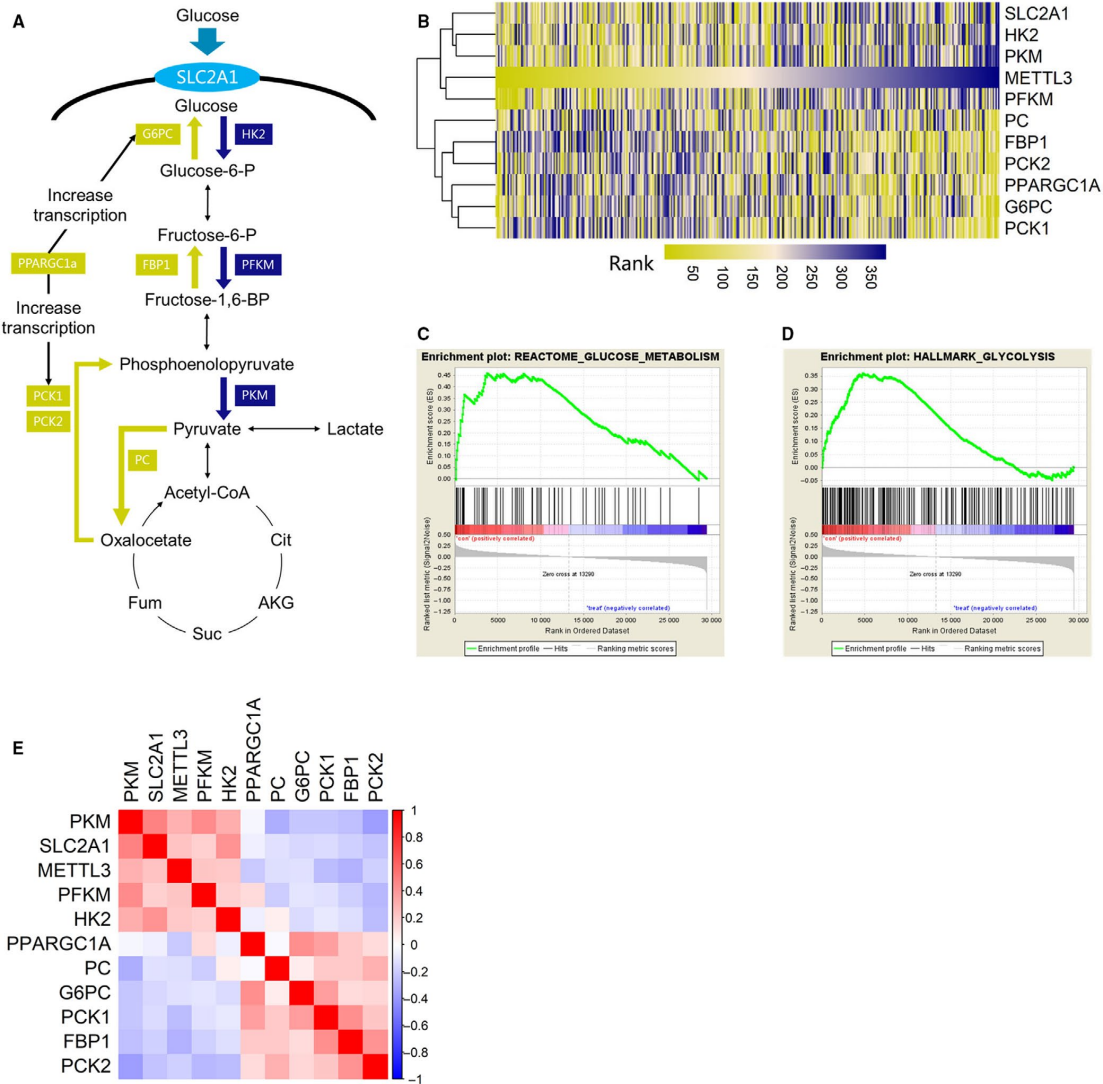
METTL3 expression was associated with altered glucose metabolism in HCC cases. A, Illustration of enzymes involved the key steps in glucose metabolic flux. B, Heatmap with cluster analysis of METT3 expression and glucose metabolism related genes revealed a co‐expression trend between METTL3 and genes involved in glycolysis. C, D, GSEA plot of two predefined gene sets based on the METTL3 expression revealed genes involved in normal glucose metabolism were significantly enriched in HCC cases with lower METTL3 expression, which suggesting that normal glucose metabolism was preserved in these cases. E, A heatmap displaying the Spearman rank correlation test results which implied positive correlation between METTL3 expression and glycolysis‐related genes and negative correlation between METTL3 expression and glycogenesis‐related genes, the bar indicates the value of spearman correlation coefficient

Due to the importance of reprogrammed glucose metabolism in tumorigenesis, we further investigate the association between METTL3 and glucose metabolism in HCC. Enzymes involved in key steps of predominant metabolic flux during glucose metabolism in mammalian cells were chosen for further investigation (Figure 3A). Overall, glucose‐6‐phosphatase catalytic subunit (G6PC), phosphoenolpyruvate carboxykinase 1 (PCK1), phosphoenolpyruvate carboxykinase 2 (PCK2), pyruvate carboxylase (PC), peroxisome proliferator‐activated receptor gamma, coactivator 1 alpha (PPARGC1A), and Fructose‐1,6‐bisphosphatase (FBP1) were selected as gene signature of gluconeogenesis pathway. In addition, glucose transporter member 1 (SLC2A1), hexokinase 2 (HK2), phosphofructokinase, muscle (PFKM), and pyruvate kinase (PKM) were selected as gene signature of glycolysis pathway.

A heatmap with cluster analysis involving the above gene signatures, together with METTL3, was plotted. As shown in Figure 3B, two main clusters were developed. One cluster was composed of genes involved in gluconeogenesis pathway, the other cluster was composed of METTL3 and genes involved in glycolysis, suggesting a highly positive correlation between expression of METTL3 and expression glycolysis genes. We further performed GSEA using TCGA data. As shown in Figure 3C,D, both two predefined gene sets, including the “REACTOME_GLUCOSE_METABOSIAM” and the “HALLMARK_GLYCOLYSIS”, were composed of genes involved in normal glucose metabolism. GSEA showed that these genes were most commonly enriched in the samples with high expression levels of METTL3 (NOM *P*‐value = .048 in REACTOME_GLUCOSE_METABOLISM; NOM *P*‐value = .045 in HALLMARK_GLYCOLYSIS). The above results suggested that normal glucose metabolism was preserved in HCC cases with low METTL3 expression. At last, the correlations between METTL3 and metabolism genes were also analyzed with spearman correlation test. As summarized in the heatmap (Figure 3E), we identified positive correlations between METTL3 and genes involved in glycolysis genes and negative correlations between METTL3 and gluconeogenesis genes.

### 3.3 METTL3 silencing in HCC cells reduces glycolysis activity

**Figure 4:**
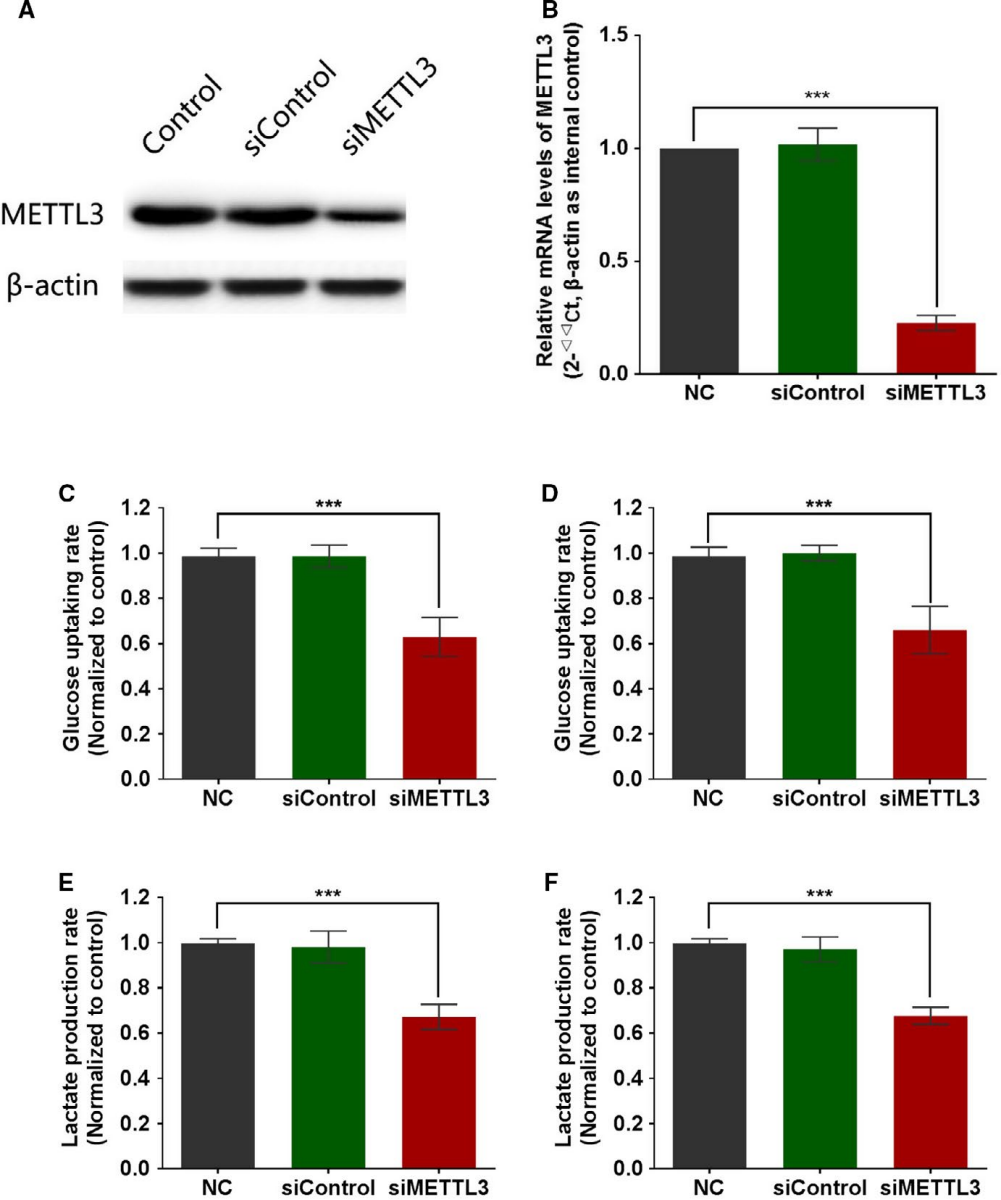
Effect of METTL3 knockdown on glycolysis activity in HCC cells. A, B, Human HCC cells Huh‐7 and SMMC‐7721 were transfected with treated with siRNA oligonucleotides, for 48 h followed by Western blot analysis (A) and qRT‐PCR (B). C, D, Human HCC cells Huh‐7 (C) and SMMC‐7721 (D) were transfected with treated with siRNA oligonucleotides for 48 h followed by glucose uptake assays. E, F, Human HCC cells Huh‐7 (E) and SMMC‐7721 (F) were transfected with treated with siRNA oligonucleotides for 48 h followed by lactate production assays

The effect of METTL3 silencing on glycolysis was further analyzed in Huh7 cells and SMMC‐7721 cells. As shown in Figure 3A,B, METTL3 in HCC cell lines, Huh‐7 and SMMC‐7721, were knockdown by transfection with siRNA oligonucleotides. The glucose uptake rate and lactate secretion rate were both significantly decreased after METTL3 silencing (Figure 4C‐F).

### 3.4 METTL3 knockdown impaired mTOR activity

**Figure 5:**
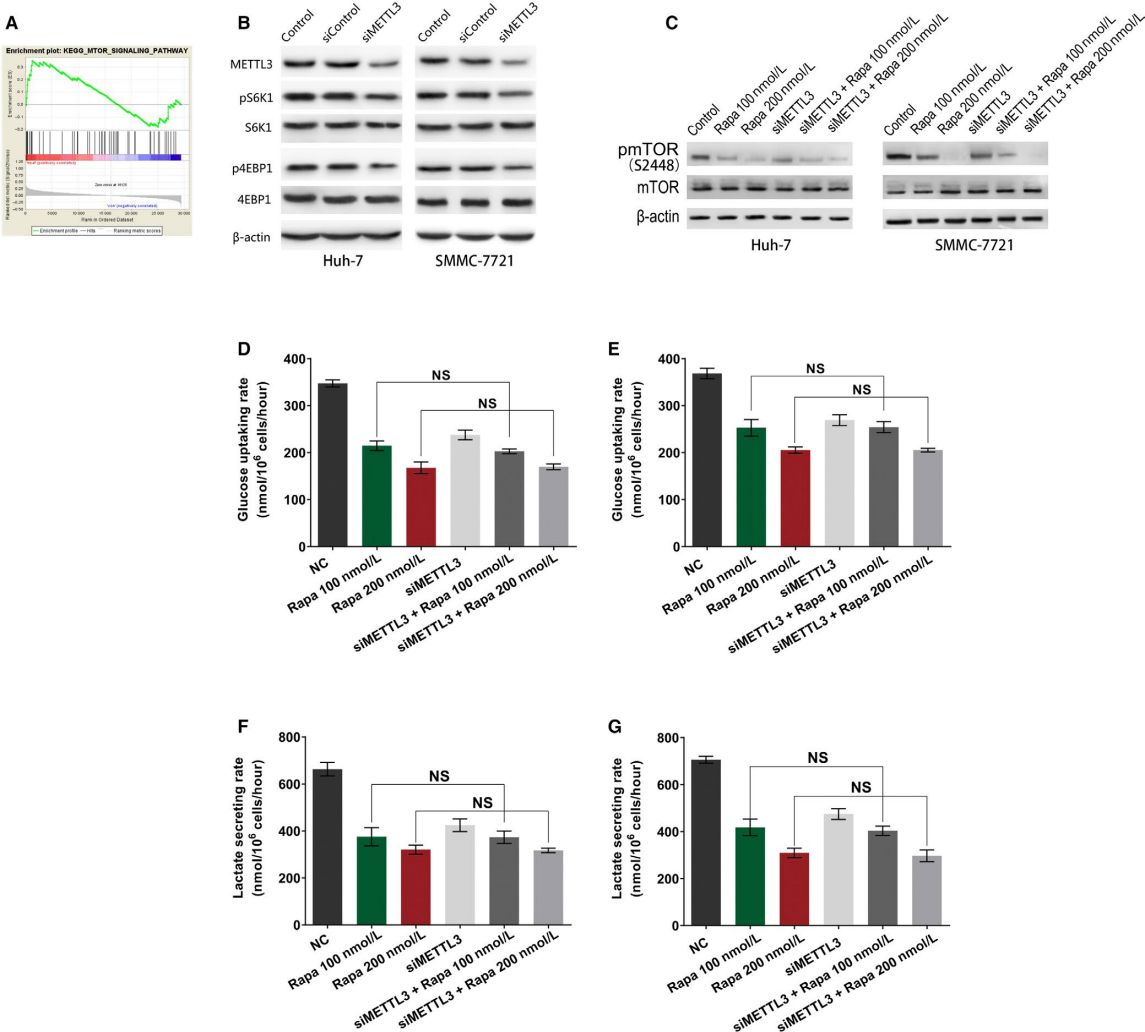
Correlation between METTL3 expression and activity of mTORC1 activity. A, GSEA plot of mTOR signal pathway based on METTL3 expression levels in TCGA cases. B, HCC cell lines were treated with siRNA oligonucleotides or not, then the indicated proteins were detected by WB. (C‐G) The HCC cell lines were treated with rapamycin or co‐treated with siRNA oligonucleotides, for 48 h followed by immunoblotting for mTOR phosphorylation levels (C), glucose uptake assays in HCC cells Huh‐7 (D) and SMMC‐7721 (E), and lactate production assays in Huh‐7 cell line (F) and SMMC‐7721 cell line (G). H, I, Gene‐specific m6A‐IP‐qPCR results showing the relative methylation levels of five RNAs in the HCC cell lines treated with siRNA oligonucleotides compared to those normal control cells

A significant GSEA enrichment score was obtained from TCGA dataset, indicating that a high enrichment in the mTOR signaling signature in the HCC cases with higher METTL3 expression (Figure 5A). Due to the importance of mTORC1 pathway in regulating glucose metabolism, we further examined the effects of METTL3 knockdown on mTORC1 activity. We found that METTL3 knockdown by siRNA decreased the mTORC1 activity as measured by S6K1 and 4EBP1 phosphorylation in both cell lines (Figure 5B).

To further evaluate whether METTL3 regulates glycolysis depends on mTOR activity, HCC cell lines were treated with rapamycin or co‐treated with siRNA oligonucleotides, after 48 hours of treatment, the phosphorylation level of mTOR, glucose uptake rate, and lactate secretion rate were examined. We found that the phosphorylated‐mTOR level was inhibited as expected after treated with rapamycin, but the phosphorylated‐mTOR level did not decreased after additional METTL3 silencing (Figure 5C). The glucose uptake rate and lactate secretion rate were significantly decreased followed by rapamycin treatment and were also not significantly changed in cells co‐treated with rapamycin and METTL3 silencing (Figure 5D‐G).

### 3.5 METTL3 silencing sensitized HCC cells to 2‐deoxyglucose

**Figure 6:**
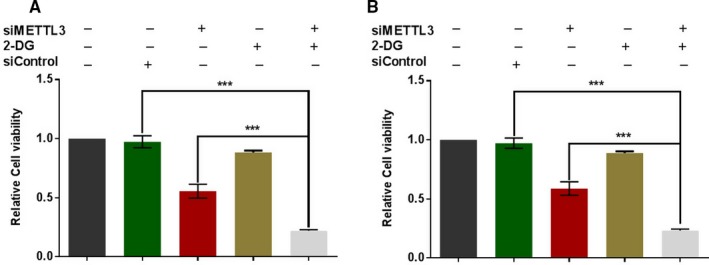
Effect on METTL3 knockdown on response of HCC cells to glycolytic stress. A, B, HCC cell lines Huh‐7 (A) and SMMC‐7721 (B) were treated with siRNA oligonucleotides, 2‐DG, or combined, then the cell viability in each group was detected by CCK‐8 assay

Given the critical association between METTL3 and glucose metabolism, we further investigated whether METTL3 knockdown could sensitize HCC cells to glycolytic stress. 2‐deoxy‐D‐glucose (2‐DG) is an analog of glucose which can induce multiple forms of metabolic stress through its action as a glycolytic inhibitor.^12^ Huh7 cells and SMMC‐7721 cells were treated with 2 mM 2‐DG. As shown in Figure 6, we found that METTL3 knockdown could suppress cell growth in both huh‐7 cells (Figure 6A) and SMMC‐772 cells (Figure 6B). In control cells without METTL3 knockdown, 2DG treatment just induced slightly sell growth suppression, but 2‐DG could induce significant inhibition of cell viability in both HCC cell lines with METTL3 knockdown (Figure 6A,B).

## 4 DISCUSSION

Here, we described that high METTL3 expression was associated a activated glycolysis phenotype in HCC samples. We found decreased METTL3 expression could inhibit the mTORC1 activity and vice versa. We also revealed that decreased METTL3 expression increased the toxicity of 2‐DG in HCC cells.

METTL3, an S‐adenosyl methionine‐binding protein with methyltransferase capacity, is the key component of the human methyltransferase complex which regulate the abundance and distribution of m6A modifications at the transcriptome level.^13^ The METTL3‐mediated m6A modifications have been proven to be involved in the development and progression of different types of cancer. Interestingly, the role of METTL3 in cancer seems to be dependent on tumor types. For example, it has been reported that METTL3 promotes ovarian carcinoma growth and epithelial to mesenchymal transition and promotes chemo‐ and radio‐resistance in pancreatic cancer cells^14^, ^15^; in contrast, METTL3 might act as a tumor suppressor in patients with renal cell carcinoma or endometrial cancer.^16^, ^17^ Despite being a m6A writer, very recent studies uncovered that METTL3 can regulate mRNA structure and subsequently promote oncogene translation independently on its m6A catalytic activity.^18^ Therefore, the mechanisms and role of METTL3 in cancer is probably very complex. Although a previous study revealed that METTL3 promoted the development of HCC,^11^ more functional roles of METTL3 in HCC remain to be determined. Our present study discovered a positive correlation between METTL3 and glycolysis metabolism in HCC cells. Additionally, we also demonstrated that METTL3 knockdown could include inhibited mTORC1 activity. These findings were in consistent with the previous report and provided evidences supporting the oncogenic role of METTL3 in HCC.^1^

TORC1 (mTOR complex 1) is a protein kinase complex that activates translation at the level of initiation and elongation. mTORC1 is a central regulator of cellular growth in response to the nutrition status.^19^ Emerging evidence indicates that mTOR may act as a controller of anabolic metabolism.^20^, ^21^ By GSEA, we found that mTORC1 signaling is positively associated with high METTL3 expression. S6K1 and 4EBP1 phosphorylation are two indicators reflecting the activity of mTORC1. Our in vitro findings showed that inhibited METTL3 expression could induce decreased phosphorylation levels of S6K1 and 4EBP1, suggesting METTL3 can lead to the activation of mTORC1 pathway. Our experiments further revealed that additional silencing of METTL3 cannot further decrease the phosphorylation level of mTORC1 and glycolysis activity in Rapamycin‐treated (mTOR‐suppressed) HCC cells. This mechanism partly explained the association between METTL3 and glycolysis metabolism in HCC. However, our study was limited by that we did not clarified the key m6A‐modification targets of METTL3, the major m6A methylase “writer” gene, to the metabolism control. Several critical matter need to be addressed in further study, including whether METTL3 directly affect the m6A modification levels in key genes responsible for glycolysis, whether METTL3 could affect the m6A modification levels of genes involved in the upstream signaling pathways responsible for metabolism regulation, and which “reader” protein mainly mediates m6A‐dependent mechanism involved in the regulation of glucose metabolism.

Through co‐expression analysis, the present study revealed a close association between METTL3 expression and transcriptional profiling of genes specific to glucose metabolism in clinical samples. One limitation of this analysis was that the transcriptional profiling did not contain information about enzyme activity and metabolic products. Therefore, we further examined the effect of METTL3 knockdown on glycolysis activity in HCC cells, and found that decreased METTL3 expression promoted the production of lactate and glucose consumption in HCC cells, representing the activation of glycolysis process due to the decreased expression of METTL3. Our studies also uncovered a synergistic effect of METTL3 KD and 2‐DG on cancer cell proliferation. This phenomenon was probably due to combination of suppressed glycolysis and METTL3 knockdown induced other downstream growth inhibition effect. In anti‐tumor therapy perspective, targeting METTL3 is of particularly promise when combined with other antimetabolites. Altogether, our studies showed that, besides the known tumor‐promoting effect in HCC, METTL3 can activate mTORC1 pathway and promote glycolysis in HCC cells. METTL3 is an ideal therapeutic target for HCC either by itself or in combination with antimetabolites.

## Data Availability

The data were available in the TCGA dataset (https://portal.gdc.cancer.gov/).
